# Multiscale Dense U-Net: A Fast Correction Method for Thermal Drift Artifacts in Laboratory NanoCT Scans of Semi-Conductor Chips

**DOI:** 10.3390/e24070967

**Published:** 2022-07-13

**Authors:** Mengnan Liu, Yu Han, Xiaoqi Xi, Linlin Zhu, Shuangzhan Yang, Siyu Tan, Jian Chen, Lei Li, Bin Yan

**Affiliations:** Henan Key Laboratory of Imaging and Intelligent Processing, PLA Strategic Support Force Information Engineering University, Zhengzhou 450001, China; liitledreanliu2022@gmail.com (M.L.); hyxxq1314@gmail.com (Y.H.); familyshuke@gmail.com (X.X.); lionelzhu96@gmail.com (L.Z.); yangshuangzhan20@gmail.com (S.Y.); siyu_tan@bjtu.edu.cn (S.T.); kronhugo@163.com (J.C.); tansiyu2011@gmail.com (L.L.)

**Keywords:** nanoCT, thermal drift, deep learning, multiscale network

## Abstract

The resolution of 3D structure reconstructed by laboratory nanoCT is often affected by changes in ambient temperature. Although correction methods based on projection alignment have been widely used, they are time-consuming and complex. Especially in piecewise samples (e.g., chips), the existing methods are semi-automatic because the projections lose attenuation information at some rotation angles. Herein, we propose a fast correction method that directly processes the reconstructed slices. Thus, the limitations of the existing methods are addressed. The method is named multiscale dense U-Net (MD-Unet), which is based on MIMO-Unet and achieves state-of-the-art artifacts correction performance in nanoCT. Experiments show that MD-Unet can significantly boost the correction performance (e.g., with three orders of magnitude improvement in correction speed compared with traditional methods), and MD-Unet+ improves 0.92 dB compared with MIMO-Unet in the chip dataset.

## 1. Introduction

NanoCT has been widely used in chemical, biomedicine, and industrial inspection [1,2,3,4]. Researchers study the internal structure of objects non-destructively with the results of 3D reconstruction. However, the focal spot drift of the X-ray source and mechanical thermal expansion can make the projections drift, which severely reduces the reconstructed resolution of nanoCT [5,6,7,8,9]. Although significant progress has been made in mechanical stability, precise alignment at the nanoscale is still stringent, so the drift phenomenon is unavoidable. Manual correction is time-consuming and laborious due to the randomness and non-repeatability of drift. Therefore, developing an effective method for the automated correction of drift artifacts has been of great interest.

Existing artifacts autocorrection is challenging. First, the drift is unpredictable [9], so the expression for drift is challenging to be explored. Second, the drift occurs throughout the scan [10], and finding a baseline to correct drift is challenging. Third, other factors (e.g., jitter, noise and brightness) render correction challenging.

Many works have been proposed to address the technical challenge. They focus on projection alignment to achieve correction. The mainstream correction methods can be divided into three categories. The first method is to add additional markers or phantoms [5,11,12]. The projections are aligned by tracking the feature location of the markers or phantoms. However, additional markers (or phantoms) may block incident X-ray. The second method aligns the original projections using the rapidly acquired sparse projections (named reference projections) as the baseline [9,10,13]. However, the method is time-consuming because of the additional scanning. In addition, the correction accuracy may be affected by temperature variations and jitter while obtaining the reference projections. The third method uses only the original projections. The method constructs the motion model of the feature points in projections and corrects the drift by the difference between the ideal model and the drift model [14,15,16,17,18]. However, the correction accuracy of the method is limited by the projection truncation and object shape. Since the existing correction methods are completely projection-dependent, the correction accuracy may be significantly reduced when the sample (e.g., chip) cannot be penetrated by X-ray at some rotation angles. Manual work is required after correction so that the angle intervals where X-ray cannot penetrate are determined, and the drifts are interpolated directly at the angles. Although the operation can reduce estimation error, the manual operation makes the methods semi-automatic.

Another idea for correct drift artifacts is to process the reconstructed slices directly. In recent years, deep learning has been successful in the deblurring field [19,20]. However, two major shortcomings limit their application in nanoCT. First, the drift artifacts are due to misaligned projections. When using reconstruction algorithms (e.g., FDK algorithm [21]) to restore CT values of 3D structures, misaligned projections can influence the periphery of the precise location. Since projection drift is random, the effect on CT values is uncertain. When viewing 2D reconstructed slices, the drift artifacts are the multi-edge representation rather than edge blurring. Second, it is difficult to construct a reconstructed slices dataset with drift artifacts; only a small number of labeled datasets can be used. A network with too many parameters may lead to overfitting and a high computational complexity [22]. Therefore, it is necessary to construct a lightweight network suitable for drift artifacts correction of nanoCT.

Herein, we propose an improved network based on the multi-input multi-output Unet (MIMO-Unet), called multiscale dense Unet (MD-Unet), for correcting undesired drift artifacts in nanoCT. We provide three versions with different parameters (MD-Unet-small, MD-Unet, and MD-Unet+). Our method can achieve the fastest (MD-Unet-small) and most accurate correction (MD-Unet+) compared to the existing methods. MD-Unet is more robust than the traditional methods. MD-Unet-small achieves optimal results with minimum parameters compared to deblurring networks.

The importance of the study is as follows: first, to the best of our knowledge, this is the first example of multi-scale Unet being applied to the drift artifacts correction in nanoCT scans of chips. Second, the proposed method takes full advantage of the potential of the multi-scale network for feature extraction. In addition, we investigate using a small number of parameters and new components (e.g., encoder/decoder, feature attention model, feature fusion block, activation function, and losses) to achieve optimal performance. Extensive experiments have verified the effectiveness of the proposed components. The main contributions of this work are as follows:

(1) We propose a deep learning framework for nanoCT drift artifacts correction, which can directly correct the reconstructed slices without processing the original projections. Compared with traditional correction methods, the proposed framework completes the correction in less than 3 s, speeding up the correction time by three orders of magnitude. In addition, the framework exhibits optimal robustness in the presence of high noise.
(2) Through the analysis of ablation experiments, we observe that some components can significantly improve the correction performance of drift artifacts. Although the components are used for drift artifacts correction in nanoCT, they may lead to new insights for image correction in other fields.
(3) In the chip dataset, the proposed network achieves superior performance compared to MIMO-Unet in fewer parameters (MD-Unet-small has 60.4% of parameters in MIMO-Unet).

## 2. Related Work

### 2.1. Drift Artifacts Correction Method Based on Reference Projections

Drift artifacts appear because of the misalignment of the original projections. Aligning the original projections with the reference projections is a commonly used method for accurate correction. The reference projections are sparse projections, which are quickly acquired after the original projections are obtained. The method was first proposed by Sasov [9] and was used to correct for thermal drift on the submicron CT systems (SkyScan scanners). The method of projection alignment is the key because it is related to the accuracy of the drift estimation. Sasov suggested using the least-squares alignment method [23]. However, when the number of reference projections is large, the correction efficiency may be reduced. The current common approaches are to use image alignment methods (e.g., ECC [24], single-step DFT algorithm [25], SURF [26], and variants of SURF [27,28]) to accelerate the correction.

**Figure 1:**
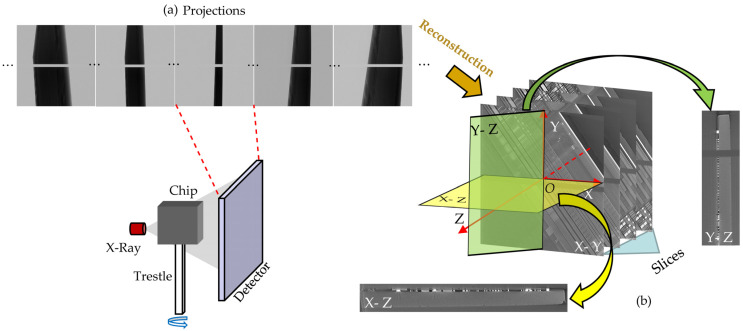
Chip scanning process and coordinate system. (**a**) Projections acquired by the detector. During the scanning process, the chip is fixed on the trestle. The trestle is rotated to obtain projections at different rotation angles. The missing data (horizontal stripe) arises from the vertical stacking of tow detectors. (**b**) 3D coordinate system for reconstructed data.

The correction framework based on reference projections is challenging to accommodate fast and robust requirements. Three main reasons are listed:

(1) Additional scanning (usually 10% of the original number of projections) is required to construct the reference projections, which can reduce correction efficiency.
(2) Manual work is required to improve correction accuracy when the reference-projection-based correction method is used to process the chips. Figure 1a shows the scanning process of the chip. Here, we highlight partial projections with missing attenuation coefficients. This happens when the chip plane is parallel to the X-ray (or in the nearby angular range). Existing projection alignment methods may degrade or even fail because the textures of projections are missing. Therefore, it is necessary to manually determine the failure interval and interpolate the estimated drift directly at the endpoints to reduce errors.
(3) When the noise increases, the correction accuracy of the correction framework based on reference projections can decrease. In some scanning tasks, it is often necessary to reduce the voltage or decrease the exposure time, which can lead to additional noise in the projections. However, the increase in noise can reduce the projection alignment accuracy and cause the drift artifacts correction to fail.

Therefore, we recommend correcting the reconstructed slices directly rather than dealing with the original projections to address the limitations. We establish a coordinate system with the reconstructed 3D data center as the origin, as shown in Figure 1b. Circuit layer slices (X-Y) containing information are fed into the network to obtain clear correction results. Compared with the existing correction methods, the noise resistance and correction performance of the proposed method are improved significantly.

### 2.2. Multiscale Network

Multiscale networks essentially embody a coarse-to-fine image correction strategy that has been applied to image deblurring [19,20,29,30] and image super-resolution tasks [31,32]. A straightforward idea is the stacking of multiple sub-networks, progressively improving the sharpness of the image from the bottom sub-network to the top sub-network. Nah et al. [20] proposed a dynamic scene deblurring (DeepDeblur), which cascaded multiple CNN networks and allowed the multiscale information flow to pass through the sub-networks, thus achieving coarse-to-fine image blur correction. However, the direct stacking of networks leads to more difficult training. Tao et al. [29] proposed a scale-recurrent network (SRN) based on the coarse-to-fine correction strategy, and they shared the information flow of the previous scale with the next scale compared to the traditional cascaded network, which makes it easier to train. However, these methods are essentially stacks of networks, and the computational complexity and memory usage are inevitably increased. In 2021, Cho et al. [19] proposed a new multiscale architecture to remove image blurring named MIMO-Unet. The method can input and output images of different scales in a single Unet, so the network parameters and training difficulty are reduced. Inspired by the success of MIMO-Unet, we construct an improved network to correct undesired drift artifacts in nanoCT. The proposed lightweight network (MD-Unet-small) reduces the parameters of MIMO-Unet by 39.6% and achieves better results compared with original MIMO-Unet. The correction results of MD-Unet+ improve by 0.92 dB in the simulation test (compared with MIMO-Unet) and achieve the sharpest result in the actual scanned chips.

## 3. Method

**Figure 2:**
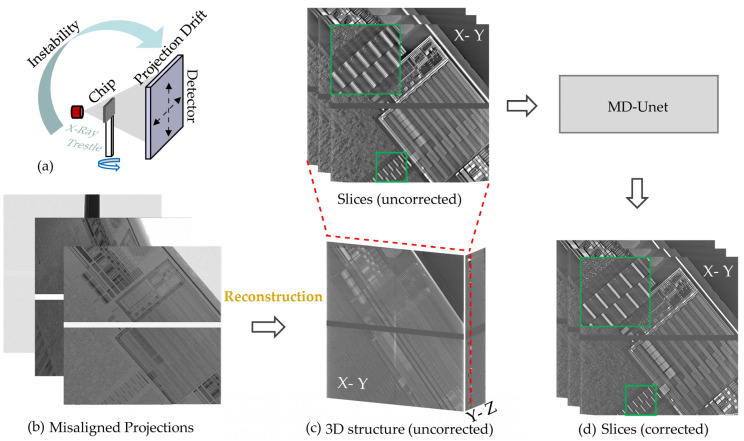
Process of the proposed correction method. The input of MD-Unet are slices (X-Y) directly reconstructed by the misaligned projections, and the sharp slices (X-Y) are outputted. (**a**) Causes of drift artifacts. (**b**) Misaligned projections obtained in unstable scan. (**c**) Uncorrected reconstructed results. (**d**) Corrected results.

The process of the proposed correction method for laboratory nanoCT is shown in Figure 2. The causes of drift are shown in Figure 2a. The temperature inside the cabinet changes continuously during the nanoCT scan. However, the slow control of the air conditioning does not keep the temperature stable, so the drift of the X-ray source and the thermal expansion of the trestle can cause the projections to drift. The FDK algorithm [21] is used to reconstruct the misaligned projections (Figure 2b). The reconstructed slices contain severe artifacts, and detailed structures cannot be distinguished (Figure 2c). The reconstructed slices with the drift artifacts are fed into MD-Unet, which suppresses the drift artifacts in the slices and produces clear results (Figure 2d).

Conceptually, the proposed correction method is the post-processing of images. The main development reported herein is applying the designed multiscale network for drift artifacts correction in nanoCT. The proposed method directly corrects the reconstructed slices without the complex process of iterations and reference baselines. In this study, we only correct the circuit layers of the chip (X-Y, the coordinate system is shown in Figure 1). We adopt the approach based on two considerations: first, the circuit layers contain more structure compared with side slices (X-Z and Y-Z). Although side slices are very important, clear imaging of circuit layers has met the needs of chip defect detection in the industry. Second, the number of circuit layers is usually small, which means that the useful information for side slices is limited.

In this section, we first introduce the proposed network architecture. Then, the multiscale mixed loss is introduced.

### 3.1. Network Architecture

**Figure 3:**
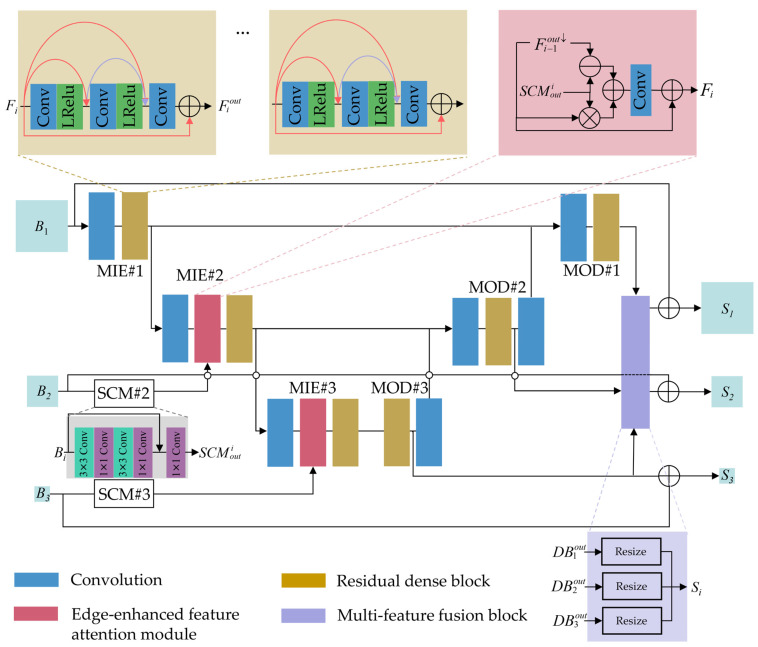
The architecture of MD-Unet. MD-Unet inputs multiscale slices containing artifacts ($B_{i}$) and generates sharp multiscale slices ($S_{i}$). ResBlocks are replaced by residual dense blocks. All non-1 × 1 convolutions are replaced by Do-Conv. MD-Unet uses the proposed EFAM and MFFB in the shallow features. SCM, MIE, and MOD are shallow convolutional module, multiscale input encoder, and multiscale output decoder, respectively. Conv and LRelu are Do-Conv and LeakyRelu, respectively.

We adopt the high-level architecture design of MIMO-Unet [19], a U-shaped network with multi-scale input and multi-scale output, and redesign the components. The proposed method consists of four parts: (1) multiscale input encoder (MIE); (2) multiscale output decoder (MOD); (3) multi-feature fusion block (MFFB); (4) multiscale mixed loss. The proposed network architecture is shown in Figure 3. Given a training sample in the uncorrected slices, the MIE encodes and fusion the adjacent-scale features (MIE#2 and MIE#3 encode and fusion; MIE#1 only encodes). We introduce a new feature attention module, named edge-enhanced feature attention module (EFAM), to emphasize edges when fusing downsampled features and reduced size features. The MOD and MFFB are then used to generate the multi-scale sharp slices.

Five main modifications are considered in the network: (1) replace the residual blocks (written as ResBlocks) with residual dense blocks; (2) replace Relu with LeakeyRelu; (3) use depthwise over-parameterized convolution (Do-conv) [33] instead of non-1 × 1 convolutional layer; (4) replace the original feature attention module (named FAM in MIMO-Unet) with the proposed EFAM; (5) replace the original asymmetric feature fusion (named AFF in MIMO-Unet) by MFFB in the shallow features.

**Residual Dense Block** Previous studies show that more layers [34,35,36,37] can improve the performance of the network. However, the direct cascade of ResBlocks makes local convolution blocks unable to access subsequent layers, and the information of all internal layers cannot be fully used [38]. Residual dense block that combines Resblock and dense connections has been proved to be superior to ResBlock in super-resolution imaging task [37]. Therefore, we consider reducing the number of cascades and deepening the network through residual dense block. We provide 3 versions, MD-Unet-small, MD-Unet, and MD-Unet+, which use 1 residual dense block, 3 residual dense blocks, and 5 residual dense blocks, respectively. In Section 5, we test the trade-off between the number of parameters and the effect of correction.

**Depthwise over-parameterized convolution** Do-Conv has shown great potential in computer vision tasks [33]. In our network, Do-Conv is used to replace all non-1 × 1 convolution. In addition, we experimentally verify the significant advantage of using Do-Conv instead of traditional convolution for result improvement.

**Edge-enhanced feature attention module** MIMO-Unet proposed a feature attention module based on element multiplication and addition used to enhance or suppress features of the previous scale [19]. The proposed EFAM focuses on the edge of the slices because uncorrected slice edges have more artifacts information. In Section 5, we verify that this module can effectively improve the performance of the network compared with the traditional fusion.

**Multi-feature fusion block in shallow features** In the general multi-scale feature fusion, large-scale features flow into the small-scale feature, which makes the feature fusion incomplete when the cross-scale increases, resulting in feature loss. Another way is to incorporate feature fusion into the network [19], but the fusion mode and the input of the decoder overlap, resulting in information redundancy. Therefore, we propose a new multi-scale feature fusion approach, which fuses the final scale information, and the outputs are a kind of mixed information so that multi-scale information can be fully integrated. We fuse the features into shallow features to output multi-scale images. The network can learn multi-scale information, and there is no waste of scale information.

### 3.2. Loss Function

We define $T_{i}$ and $S_{i}$ as ground truth and generated sharp slices on the *i* th scale, respectively. To train MD-UNet, three loss functions are used:

(1) Multi-scale content (MSC) loss:

(1) $$L_{MSC}={\sum_{i}\left\|S_{i}-T_{i}\right\|}_{1},$$

(2) Multi-scale edge (MSED) loss, which is used to evaluate the difference between the generated image and the ground truth on the edge to enhance the expression of the network in detail:

(2) $$L_{MSED}=\sum_{i}\sqrt{{\left\|\Delta S_{i}-\Delta T_{i}\right\|}^{2}+\varepsilon },$$

where Δ is the Laplacian operator, ε is a constant value $10^{-6}$.

(3) Multi-scale frequency reconstruction (MSFR) loss, which is used to constrain the consistency between the generated image and the ground truth in the frequency domain:

(3) $$L_{MSFR}={\sum_{i}\left\|F(S_{i})-F(T_{i})\right\|}_{1},$$

where *F* is the FFT transalation.

Finally, the multiscale mixed loss (L) in the MD-Unet can be expressed as:

(4) $$L=L_{MSC}+\alpha L_{MSED}+\beta L_{MSFR,}$$

where α and β are set as 0.6 and 0.1.

## 4. Experiment

### 4.1. Dataset and Implementation Details

In order to verify the effectiveness of our method on drift artifacts correction, we establish a dataset of chip slices. Magnification ratio and exposure time are adjusted to mitigate the effects of drift on projections, and reconstructed slices are used as labels. The actual nanoCT drifts measured by previous experiments are added to the projections to generate reconstructed slices with drift artifacts. The drifts continuously vary and range from −15 pixels to 15 pixels. The training set is expanded to 1100 pairs of slices through data augmentation, which has three ways:

(1) Cropping, 0–2% of the edge is cropped with a probability of 0.5.
(2) Scaling, the scaling ratio is 99–101%.
(3) Translation and rotation, the translation range is from −1 pixel to 1 pixel, and the rotation range is from −5° to 5°.

The validation set consists of 55 pairs of slices that do not participate in training. The test chips are divided into simulation verification (one chip is shown, named Chip 1) and actual correction (two chips are shown, named Chip 2 and Chip 3). The scan parameters are listed in Table 1. Chip 1 is scanned at low magnification and short exposure time, so we add the drift from the previous measurement for simulation verification. Part of a bee mouth was scanned in the previous scan. Since the bee mouthpart is not a flat sample (X-ray can penetrate at all rotation angles), the reference-projection-based correction method was used to estimate drift. The discrete Fourier transform (DFT) of the projection is used to achieve sub-pixel translation. Chip 2 and Chip 3 are scanned at high magnification for actual correction. The nanoCT operates at 60 kV. Chip 2 is scanned at a full angle with an exposure time of 15 s and a rotation step of 0.36°. The reference projections are acquired in 3.6° steps because the traditional correction methods are used as the contrast. The full-angle projections of Chip 3 are acquired with 3° steps and 15 s exposure time.

We train our network for 600 epochs, which can make the net fully learn drift artifacts features. The batch size is 10. The learning rate is set at 0.0001. Our experiments are performed on Xeon Gold 5118CPU (128GB) and NVIDIA GeForce RTX 2080Ti (×4).

### 4.2. Experiments Setting

First, the proposed method is evaluated by Chip 1.

Second, the actual scanned Chip 2 (scan parameters are listed in Table 1) is used to evaluate the correction effectiveness of the proposed method and the traditional methods. The chip had been shown in the previous study [17] but on different layers. The proposed method is compared with the mainstream methods for nanoCT drift artifacts correction, which are reference-projections-based methods. ECC [24] and single-step DFT algorithm [25] use projection intensity to align projections. LPM [27] and RANSAC [28] are variants of SURF that eliminate outliers from the original features (extracted by SURF) to achieve accurate projection alignment. The number of iterations for ECC is 5000, the upsampling factor for DFT is 1000, and the threshold for SURF is 200. To evaluate the robustness of the correction methods, we add additional noise to the projections of Chip 2. The added noise follows the Poisson distribution to simulate detector noise [39], and three noise levels (5%, 10%, and 15%) are considered. Here, the noise level is defined as the ratio of the noise sum to the sum of the projection.

Third, the proposed network is compared with the mainstream and the latest Deblurgan-v2 [30] and MIMO-UNet [19]. To train fairly, we keep the parameters suggested by the authors and train the network until convergence. Three versions of MD-Unet are provided: MD-Unet-small, MD-Unet, and MD-Unet+ have a number of dense residual blocks of 1, 3, and 5, respectively. Chip 1 is used to test the networks to evaluate the difference between the correction results and the ground truth. The actual scanned Chip 3 is used to show the correction effects of different networks. In addition, ablation experiments are considered to evaluate the effectiveness of the proposed components. Five components of the architecture in MD-Unet are evaluated: (1) encoder/decoder. MD-Unet uses dense residual blocks (with Do-Conv). Here, we evaluate two other forms, dense residual blocks (without Do-Conv) and ResBlock (8 stacked ResBlocks that are used in MIMO-Unet). (2) Activation function. LeakeyRelu is used in MD-Unet. We evaluate Relu to demonstrate the positive effect of LeakeyRelu on results. (3) Feature attention. Traditional element multiplication, element addition, and FAM used in MIMO-UNet are considered to demonstrate the advantages of the proposed EFAM. (4) Multi-scale feature fusion. MIMO-Unet fuses multi-scale features upon completion of coding (this module is named FAM in their work). Here, we evaluate the proposed MFFB (in shallow features) with FAM and without fusion. (5) Loss. Three different combinations of losses are evaluated (MSC, MSC+MSED, and MSC+MSRF).

## 5. Results and Discussion

### 5.1. Evaluation of Drift Artifacts Correction on Chips

**Figure 4:**
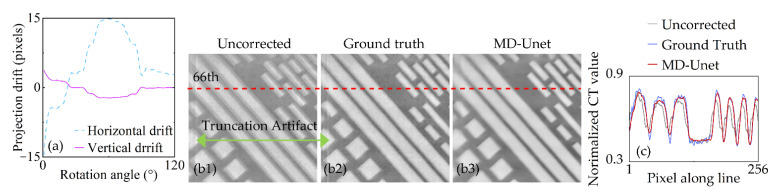
Reconstructed slice and profile of Chip 1. (**a**) Drift added to the projections. (**b1**) Uncorrected slice. (**b2**) Ground truth. (**b3**) Correction results of the proposed method. (**c**) Profiles of the 66th line.

First, we test the proposed method by the simulated artifacts, and the result is presented in Figure 4. Figure 4a shows the drift added to the projections of Chip 1, which is a continuous and stable process. Figure 4b1–b3 shows the uncorrected slice, ground truth, and corrected slice of the proposed method (MD-Unet), respectively. The uncorrected slice has multi-edge due to the unaligned projections. In addition, projection truncation during scanning introduces truncation artifacts, as shown by the green arrows in Figure 4b1,b2. On the one hand, the proposed method successfully corrects the drift artifacts and faithfully maintains the slice details of ground truth. On the other hand, the convolution layers in the network have the smoothing effect on the truncation artifacts and noise of the slice. We show the 66th lines of the slice profiles in Figure 4b1–b3. The results (Figure 4c) show that the details of the corrected slice are quite close to the ground truth, and the noise in the slices (Figure 4b1,b2) is effectively suppressed.

**Figure 5:**
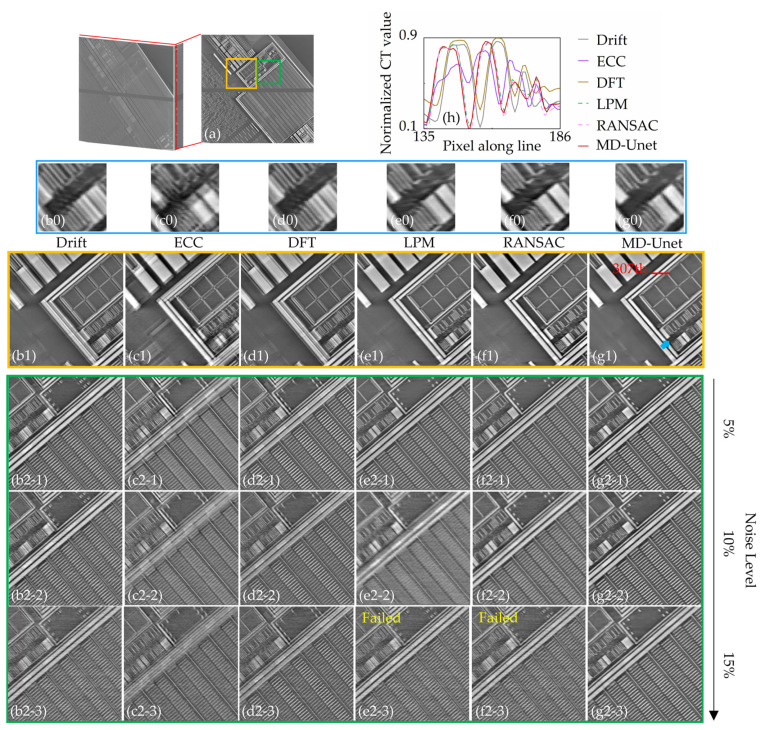
Drift artifacts correction of Chip 2. The chip is used in Ref. [13] to evaluate the reference projection correction. Here, we show the same chip but a different layer. (**a**) 3D structure of the chip, the slice of the 373rd layer, is shown. Yellow box and green box mark two local areas, which are shown in (**b0**–**b2-3**) to (**g0**–**g2-3**). Here, we only consider the methods of nanoCT drift correction. (**b0**–**g0**) are the local magnification of the area marked by the blue arrow (in (**g1**)). (**b1**–**g1**) show the local magnification of the yellow box marked in (**a**). (**b2**–**g2**) show the local magnification of the green box under three noise levels. LPM and RANSAC fail at the 15% noise level, so we fill the uncorrected slice (**b2-3**) into (**e2-3**) and (**f2-3**). (**h**) is the local profile of the 307th row marked in (**g1**).

We validate the proposed method in Chip 2 with the mainstream correction methods as the comparison, and the results are presented in Figure 5. Figure 5a shows the 72nd layer (the number of layers of the silicon substrate is considered) of the reconstructed result. Figure 5b1–g1 are the local magnification of the yellow box marked in Figure 5a, and they are the uncorrected slice, the ECC-corrected slice, the DFT-corrected slice (the single-step DFT algorithm is simply represented as DFT here, and similarly in Figure 5), the LPM-corrected slice, the RANSAC-corrected slice, and the slice corrected by our method (MD-Unet), respectively. Since drifts cause projections to move randomly (left/right or up/down), slices reconstructed from the unaligned projections contain multi-edge artifacts, which are distinguished from geometric artifacts and blurring, as shown in Figure 5b1. ECC (Figure 5c1) and DFT (Figure 5d1) do not achieve the desired correction. LPM and RANSAC eliminate the SURF outliers and achieve accurate corrections using valid projection features; their results (Figure 5e1,f1) show sharp edges and clear structures. The proposed method (Figure 5g1) successfully corrects for drift artifacts, and the correction results are as clear as those of the SURF variants (Figure 5e1,f1). However, we find that the traditional correction methods could not completely correct the drift artifacts, especially the details (marked by the blue arrow in Figure 5g1). To clearly show the detailed information of the slice, we enlarge the area marked by the blue arrow, and the results are shown in Figure 5b0–g0. The results indicate that drift artifacts make the relationship of components in the chip difficult to discern, which is very important in industry. Although LPM and RANSAC perform well in the overall correction, they are insufficiently processed in the detail part. The proposed method achieves the clearest results. Further, the local profile of the reconstructed slice, which is marked in Figure 5g1, is used to assess the correction effect. The profiles (Figure 5h) show that the DFT achieves a limited correction result, and the ECC even makes the details more indistinguishable. The profile of the proposed method is close to that of the SURF variants. It is worth noting that the proposed method does not require additional scanning, which means that the proposed method saves at least 1500 s (the time of addition scanning).

The slices used for training and testing are derived from experimental measurements and include noise associated with the actual nanoCT. However, to further evaluate the robustness of the proposed method, we add different levels of noise (5%, 10%, 15%) to the projections. Noise drowns out the detailed structure of projections, which degrades the performance of traditional projection-based methods. The area marked by the green box in Figure 5a is used to show the correction results of the different methods, as shown in Figure 5b2–g2. The increase in noise reduces the correction accuracy of traditional methods. This is well understood because the noise makes the details of the projections lost, which makes it difficult to align the projections accurately with traditional methods. It is worth noting that the SURF-extracted features are inaccurate and are completely eliminated by LPM and RANSAC in the case of 15% noise, so the LPM and RANSAC corrections fail. We fill the uncorrected results (Figure 5b2-3) into the correction results for LPM and RANSAC, respectively (Figure 5e2-3,f2-3). The results of the proposed method under different noise levels (Figure 5g2-1–f2-3) show that sharp edges and convolution layers are effective for noise removal. The results also show that the proposed method can robustly correct drift artifacts even under high noise conditions.

### 5.2. Network Performance Comparison and Ablation Experiments

We compare the proposed method with the deblurring network (Deblurgan-v2 and MIMO-Unet). Considering the tradeoff between the number of calculated parameters and accuracy, we evaluate three variants of MD-Unet: MD-UNet-small, MD-Unet, and MD-Unet+.

PSNR, SSIM, Runtime, and Params of different networks are shown in Table 2, and the best results are bolded. MD-Unet+ has the slowest runtime compared to MD-UNet-small and MD-Unet but completes the correction within 0.15 s (time to process one layer). MD-Unet+ achieves the optimal correction, and MD-Unet-small achieves the shortest correction time. MD-Unet-small, MD-Unet, and MD-Unet+ demonstrate the optimal tradeoff between computational complexity and accuracy. The proposed network achieves optimal results compared to the traditional framework of deblurring. Compared to MIMO-Unet, the proposed MD-Unet-small achieves better results with a 39.6% reduction in the number of parameters. The reconstruction sizes of Chip 2 and Chip 3 are 1065 × 1030 × 12 pixels and 1065 × 1030 × 10 pixels (silicon substrates are not considered). When using the network to correct Chip 2 and Chip 3, 12 and 10 slices are input, respectively. Therefore, the total correction times MD-Unet-small, MD-Unet, and MD-Unet+ are 0.990 s, 2.134 s, and 2.398 s. For the traditional correction methods, the correction time needs at least 1680 s in total. Therefore, the correction speed of the proposed method is improved by three orders of magnitude at least.

**Figure 6:**
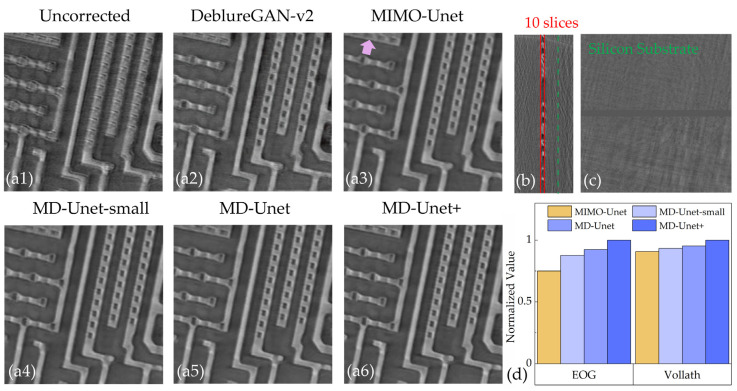
Correction results of different networks in Chip 3. (**a1**) is the uncorrected slice. (**a2**–**a6**) show corrected results of different networks. (**b**) is the side slice (Y-Z) of Chip 3. The area indicated by red lines is the circuit layers containing the information. The green dotted line marks one of the silicon substrates. (**c**) Silicon substrate. (**d**) Numerical evaluation of correction results.

Figure 6 shows the correction results for Chip 3. The uncorrected result (Figure 6a1) shows a low-resolution result, which is extremely serious because the connections of the various parts of the chip cannot be identified. Compared with the uncorrected slice, the result of DeblurGAN-v2 (Figure 6a2) is improved, but the structure and connectivity of the chip are still unclear. MIMO-Unet achieves a clear result on the overall structure, but the details are blurred. The proposed method handles the details well, especially MD-Unet+ (Figure 6a6), which achieves the sharpest result. The proposed method directly corrects 2D slices of the chip. Figure 6b shows the side of Chip 3, 10 slices containing useful information, and the other layers on the side are silicon substrates (Figure 6c). Further, we evaluate the corrected slices by image quality metrics. The energy of gradient (EOG) and Vollath function are considered. EOG contains the gradient information of the image, which is represented as:

(5) $$EOG=\sum_{M}\sum_{N}{\left[f\left(x+1,y\right)-f\left(x,y\right)\right]}^{2}+{\left[f\left(x,y+1\right)-f\left(x,y\right)\right]}^{2},$$

where f(x,y) is the 2D slice of the chip. *M* and *N* are the length and width of the slice, respectively. The gradient for the sharp slice is larger compared to the incompletely corrected result. Therefore, the sharp slice has a larger EOG. Vollath function is a classic image quality evaluation criterion, which is expressed as:

(6) $$Vollath=\sum_{M}\sum_{N}f\left(x,y\right)\left|f\left(x+1,y\right)-f\left(x+2,y\right)\right|.$$

Vollath function reflects the correlation of pixels. The sharp slice has a low pixel correlation, so the Vollath value is larger. The numerical evaluation results of Figure 6a3–a6 are shown in Figure 6d. Since the numerical ranges of EOG and Vollath do not match, normalized results are shown. The numerical evaluation results of Chip 3 are consistent with the results shown in Table 2; i.e., the proposed method outperforms MIMO-Unet in chip drift artifacts correction.

We evaluate the validity of the proposed components in the MD-Unet architecture by ablation experiments. PSNR, SSIM, and Params for networks with different components are shown in Table 3. Firstly, different encoders/decoders are evaluated. We consider two forms (residual dense blocks without Do-Conv and a stack of eight ResBlocks). Res Dense block (without Do-Conv) achieves better results than ResBlock, and the introduction of Do-Conv further improves the PSNR results by 0.32 dB. Secondly, we test the activation function, and the results show that using LeakeyRelu instead of Relu improved PSNR by 0.27 dB. Thirdly, we evaluate different types of feature-attention models (element sum, element multiplication, and FAM used in MIMO-Unet). EFAM is used in MD-UNet, and the optimal effect is achieved. Fourthly, we test the AFF used in MIMO-Unet and without feature fusion. In the proposed version, we use MFFB in the shallow features. The test results in Table 3 show that the proposed MFFB is most effective in shallow features compared to other fusion methods. Finally, different losses are assessed. The results in Table 3 show that the information in the frequency domain and edges can enhance the correction results. The proposed loss (MSC+MSED+MSRF) achieves the optimal results.

**Figure 7:**
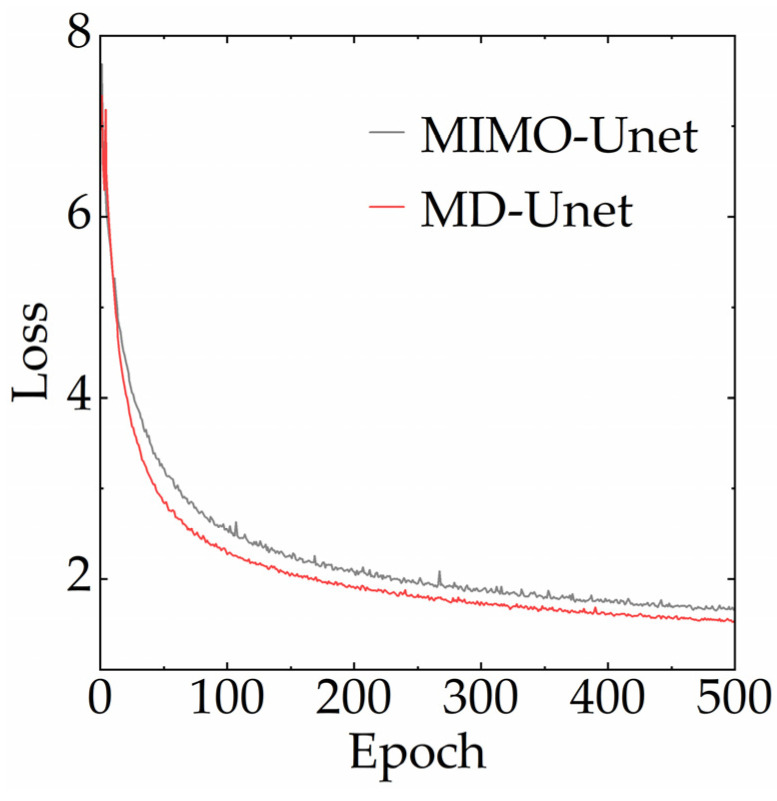
Training loss curves. MIMO-Unet and MD-Unet use the same loss, which is proposed in Section 3.2.

In addition, we evaluate the advantages of the proposed framework by recording the losses during the training process (500 epochs), as shown in Figure 7. Here, both MIMO-Unet and MD-Unet use the proposed multiscale mixed loss (Section 3.2). The results show that MD-Unet has a lower loss.

## 6. Conclusions

This paper presents a deep-learning-based method for the correction of drift artifacts in nanoCT, which directly corrects reconstructed slices without additional reference projections. The technique is based on the overall architecture of MIMO-Unet and achieves optimal performance compared to the existing methods. The test results show that the proposed network can correct undesired drift artifacts and smooth truncation artifacts and noise. In addition, the correction results for different noise levels show that the network is not sensitive to noise.

This study provides a convenient and fast drift artifacts correction method, which has two advantages compared to the traditional correction methods. First, the correction efficiency of the work is high because the network directly corrects the 2D reconstructed slices, which does not require reference projections, and determines angle intervals where attenuation coefficients are missing. Second, this work achieves stable and optimal correction results.

It should be noted that the proposed technique has a limitation. The proposed network is developed for high-quality imaging of chips. Correction performance may decrease when the test samples are of other species (e.g., plant and animal tissue). We recommend that users use the small training set for transfer learning in this case.

Overall, our method is efficient and effective. Any additional correction phantoms or reference scans are not required. After correction, the drift artifacts in the reconstructed slices are significantly suppressed. Our network is designed for correcting the drift artifacts of chips in laboratory nanoCT, but it can also be used in artifacts correction for other devices.

## Figures and Tables

**Table 1:** Scanning parameters of the chips used to test the networks.

Test Type	Chip Number	Scan Parameters					
		Voltage (kV)	Detector Size (Pixels)	Exposure Time (s)	Rotation Step of Round 1 (°)	Rotation Step of Round 2 (°)	
Simulation Verification	Chip 1	60	1065 × 1030	5	3		
	
Actual Correction	Chip 2			15	0.36	3.6	
	Chip 3			15	3		
	

**Table 2:** The average PSNR and SSIM of different networks. We employ the standard version (8 stacked Resblocks) for MIMO-Unet. The runtime and parameters are in seconds and millions, respectively. Here, PSNR and SSIM are the average correction results in the simulation experiment (Chip 1, which has ground truth). Runtime is the average time to process a slice in all experiments (Chip 1, 2, and 3). The runtime is in seconds (s), and the parameters during the training process are in millions (M). The best results are **bold**.

Model	PSNR	SSIM	Runtime (s)	Params (M)
DeblurGAN-v2	24.946	0.856	0.137	11.70
MIMO-Unet	31.775	0.970	0.075	6.81
MD-Unet-small	31.891	0.970	**0.045**	**4.11**
MD-Unet	32.155	0.972	0.097	10.55
MD-Unet+	**32.691**	**0.974**	0.109	16.98

**Table 3:** Ablation experiments for MD-Unet architecture. Here, ResBlock represents the stack of 8 ResBlocks, which is used in the standard version of MIMO-Unet. FAM and AFF are the feature attention model and multiscale feature fusion method used in MIMO-Unet, respectively. Here, PSNR and SSIM are the average results in the slices of Chip 1 (Chip 1, which has ground truth).

Block	Name	PSNR	SSIM	Param (M)
Encoder/Decoder	Res Dense Block (no Do-Conv)	31.835	0.970	8.88
	ResBlock	31.640	0.970	7.10
Activation function	Relu	31.885	0.972	10.55
Feature attention	Element sum	31.820	0.970	10.55
	Element multiplication	31.582	0.969	10.55
	FAM	31.925	0.972	10.55
Multi-scale feature fusion	AFF	32.057	0.971	10.63
	Without	31.958	0.971	7.07
Loss	MSC	31.674	0.968	10.55
	MSC+MSRF	31.917	0.970	10.55
	MSC+MFE	31.695	0.968	10.55

## Data Availability

The data and the code used for the manuscript are available for researchers on request from the corresponding author.

## References

[1] Kampschulte M., Langheinirch A.C., Sender J., Litzlbauer H.D., Althöhn U., Schwab J.D., Alejandre-Lafont E., Martels G., Krombach G.A. (2016). Nano-Computed Tomography: Technique and Applications. RoFo Fortschritte Gebiete Rontgenstrahlen Nuklearmedizin.

[2] Langheinrich A.C., Yeniguen M., Ostendorf A., Marhoffer S., Kampschulte M., Bachmann G., Stolz E., Gerriets T. (2010). Evaluation of the middle cerebral artery occlusion techniques in the rat by in-vitro 3-dimensional micro- and nano computed tomography. BMC Neurol..

[3] Su Z., Decencière E., Nguyen T.T., El-Amiry K., Andrade V.D., Franco A.A., Demortière A. (2022). Artificial neural network approach for multiphase segmentation of battery electrode nano-CT images. NPJ Comput. Math..

[4] Tjaden B., Lane J., Brett D., Shearing P.R. (2017). Understanding transport phenomena in electrochemical energy devices via X-ray nano CT. J. Phys. Conf. Ser..

[5] Vavrik D., Jandejsek I., Pichotka M. (2016). Correction of the X-ray tube spot movement as a tool for improvement of the micro-tomography quality. J. Instrum..

[6] Borges de Oliveira F., Porath M., Camargo Nardelli V., Arenhart F., Donatelli G. (2014). Characterization and Correction of Geometric Errors Induced by Thermal Drift in CT Measurements. Key Eng. Mater..

[7] Guizar-Sicairos M., Diaz A., Holler M., Lucas M.S., Menzel A., Wepf R.A., Bunk O. (2011). Phase tomography from x-ray coherent diffractive imaging projections. Opt. Express.

[8] Odstrčil M., Holler M., Raabe J., Guizar-Sicairos M. (2019). Alignment methods for nanotomography with deep subpixel accuracy. Opt. Express.

[9] Sasov A., Liu X., Salmon P. (2008). Compensation of mechanical inaccuracies in micro-CT and nano-CT. Proc SPIE.

[10] Salmon P., Liu X., Sasov A. (2009). A post-scan method for correcting artefacts of slow geometry changes during micro-tomographic scans. J. X-Ray Sci. Technol..

[11] Hiller J., Maisl M., Reindl L. (2012). Physical characterization and performance evaluation of an x-ray micro-computed tomography system for dimensional metrology applications. Meas. Sci. Technol..

[12] Vogeler F., Verheecke W., Voet A., Kruth J.P., Dewulf W. Positional Stability of 2D X-ray Images for Computer Tomography. Proceedings of the International Symposium on Digital Industrial Radiology and Computed Tomography (DIR 2011).

[13] Liu M., Han Y., Xi X., Tan S., Chen J., Li L., Yan B. (2021). Thermal Drift Correction for Laboratory Nano Computed Tomography via Outlier Elimination and Feature Point Adjustment. Sensors.

[14] Gürsoy D., Hong Y.P., He K., Hujsak K., Yoo S., Chen S., Li Y., Ge M., Miller L.M., Chu Y.S. (2017). Rapid alignment of nanotomography data using joint iterative reconstruction and reprojection. Sci. Rep..

[15] Larsson E., Gürsoy D., De Carlo F., Lilleodden E., Storm M., Wilde F., Hu K., Müller M., Greving I. (2019). Nanoporous gold: A hierarchical and multiscale 3D test pattern for characterizing X-ray nano-tomography systems. J. Synchrotron Radiat..

[16] Fu T., Zhang K., Wang Y., Li J., Zhang J., Yao C., He Q., Wang S., Huang W., Yuan Q. (2021). Deep-learning-based image registration for nano-resolution tomographic reconstruction. J. Synchrotron Radiat..

[17] Liu M., Han Y., Xi X., Zhu M., Zhu L., Song X., Kang G., Yang S., Li L., Yan B. (2021). Horizontal Drift Correction by Trajectory of Sinogram Centroid Fitting for Laboratory X-ray Nanotomography.

[18] Nikitin V., Andrade V.D., Slyamov A., Gould B.J., Zhang Y., Sampathkumar V., Kasthuri N., Gürsoy D., Carlo F.D. (2021). Distributed Optimization for Nonrigid Nano-Tomography. IEEE Trans. Comput. Imaging.

[19] Cho S.J., Ji S.W., Hong J.P., Jung S.W., Ko S.J. (2021). Rethinking Coarse-to-Fine Approach in Single Image Deblurring. arXiv.

[20] Nah S., Kim T.H., Lee K.M. (2016). Deep Multi-Scale Convolutional Neural Network for Dynamic Scene Deblurring.

[21] Feldkamp L.A., Davis L.C., Kress J.W. (1984). Practical cone-beam algorithm. J. Opt. Soc. Am. A.

[22] Wei J., Zhu G., Fan Z., Liu J., Rong Y., Mo J., Li W., Chen X. (2022). Genetic U-Net: Automatically Designed Deep Networks for Retinal Vessel Segmentation Using a Genetic Algorithm. IEEE Trans. Med. Imaging.

[23] Ackermann T.F. (2006). digital image correlation: Performance and potential application in photogrammetry. Photogramm. Rec..

[24] Georgios D., Evangelidis, Emmanouil Z., Psarakis (2008). Parametric image alignment using enhanced correlation coefficient maximization. IEEE Trans. Pattern Anal. Mach. Intell..

[25] Manuel Guizar-Sicairos S.T.T., Fienup J.R. (2008). Efficient subpixel image registration algorithms. Opt. Lett..

[26] Bay H., Ess A., Tuytelaars T., Gool L.V. (2008). Speeded-Up Robust Features (SURF). Comput. Vis. Image Underst..

[27] Ma J., Zhao J., Jiang J., Zhou H., Guo X. (2019). Locality Preserving Matching. Int. J. Comput. Vis..

[28] Torr P., Zisserman A. (2000). MLESAC: A New Robust Estimator with Application to Estimating Image Geometry. Comput. Vis. Image Underst..

[29] Tao X., Gao H., Wang Y., Shen X., Wang J., Jia J. Scale-recurrent Network for Deep Image Deblurring. Proceedings of the 2018 IEEE/CVF Conference on Computer Vision and Pattern Recognition.

[30] Kupyn O., Martyniuk T., Wu J., Wang Z. DeblurGAN-v2: Deblurring (Orders-of-Magnitude) Faster and Better. Proceedings of the 2018 IEEE/CVF Conference on Computer Vision and Pattern Recognition.

[31] Wu H., Ni N., Zhang L. (2021). Scale-Aware Dynamic Network for Continuous-Scale Super-Resolution. arXiv.

[32] Li J., Fang F., Mei K., Zhang G. Multi-scale Residual Network for Image Super-Resolution. Proceedings of the ECCV.

[33] Cao J., Li Y., Sun M., Chen Y., Lischinski D., Cohen-Or D., Chen B., Tu C. (2020). DO-Conv: Depthwise Over-parameterized Convolutional Layer. IEEE Trans. Image Processing.

[34] Zhang Y., Tian Y., Kong Y., Zhong B., Fu Y. Residual Dense Network for Image Super-Resolution. Proceedings of the IEEE Conference on Computer Vision and Pattern Recognition.

[35] Zhang Y., Li K., Li K., Wang L., Zhong B., Fu Y. Image Super-Resolution Using Very Deep Residual Channel Attention Networks. Proceedings of the ECCV.

[36] Lim B., Son S., Kim H., Nah S., Lee K.M. Enhanced Deep Residual Networks for Single Image Super-Resolution. Proceedings of the 2017 IEEE Conference on Computer Vision and Pattern Recognition (CVPR).

[37] Wang X., Yu K., Wu S., Gu J., Liu Y., Dong C., Loy C.C., Qiao Y., Tang X. (2018). ESRGAN: Enhanced Super-Resolution Generative Adversarial Networks.

[38] Mao X., Liu Y., Shen W., Li Q., Wang Y. (2021). Deep Residual Fourier Transformation for Single Image Deblurring. arXiv.

[39] Xin J., Liang L., Le S., Chen Z. (2012). Improved total variation based CT reconstruction algorithm with noise estimation. Spie Optical Engineering + Applications.

